# Electrocardiographic Analysis of Repolarization Changes in South Indian Children with Kawasaki Disease after the Acute Phase of Illness

**DOI:** 10.1155/2018/1062154

**Published:** 2018-07-02

**Authors:** Siddhartha Reddy, Maneesh Rai, Ravi Raj Singh Chouhan, Suchetha Rao, Nutan Kamath

**Affiliations:** ^1^Fernandez Hospital, Hyderabad, Telangana 500034, India; ^2^Department of Cardiology, Kasturba Medical College, Manipal Academy of Higher Education, Mangalore, Karnataka 575001, India; ^3^Kasturba Medical College, Manipal Academy of Higher Education, Mangalore, Karnataka 575001, India; ^4^Department of Paediatrics, Kasturba Medical College, Manipal Academy of Higher Education, Mangalore, Karnataka 575001, India

## Abstract

**Background:**

Cardiac involvement in children with Kawasaki disease (KD) may present with repolarization abnormalities which are associated with increased risk of ventricular arrhythmias and sudden cardiac events.

**Methods:**

Twenty children with history of KD without cardiac involvement in the acute phase were recruited along with age and sex-matched controls. Twelve-lead ECG was obtained from both groups using CARDIART 610T ECG system at 25 mm/sec and 50 mm/sec paper speed. ECG was repeated in 19 children in the study group after 9 ± 2 months. Measurements (QT dispersion (QTd), T-wave peak to end (Tp-Te) interval, and Tp-Te/QT ratio) were made using standard digital calipers. Statistical analysis was performed with student *t*-test and analysis of variance (ANOVA) using SSPS version 17.0.

**Results:**

The mean value of QTd in the first ECG in cases was significantly high: 43.15 ± 14.13 versus 29.47 ± 8.637 in the controls (*p* = 0.001). The follow-up ECG in 19 cases showed a mean value of 46.26 ± 16.25 versus 43.89 + −14.53 at baseline (*p* = 0.440). QTd was increased in the follow-up ECG but was not statistically significant. There was no statistical significance seen in the Tp-Te interval and Tp-Te/Qt ratio as observed in Lead II and Lead V5.

**Conclusion:**

Significant increase in the QTd in children with KD indicates repolarization changes in the myocardium even in the absence of clinical carditis. The persistence of this change on follow-up could indicate a possible increased risk for ventricular arrhythmia and warrants long term assessment of the cardiovascular status.

## 1. Introduction

Kawasaki disease (KD) is an acute, self-limiting, febrile illness that occurs predominantly in children with pathology demonstrating vasculitis of small and medium size blood vessels with a predilection for coronary arteries [1]. It is complicated by either clinical or subclinical myocarditis in the acute stage and can lead to histological changes of the myocardium such as interstitial fibrosis. These abnormalities may persist even after the acute phase without the involvement of the coronary arteries [2].

Myocardial damage results in altered electrical potential distributions and repolarization changes manifesting as prolonged Qt dispersion and electrophysiological changes in T-waves [3, 4].

T peak is a marker of epicardial repolarization while T end is believed to represent completion of repolarization of the mid-myocardial cells. The interval between T peak and T end provides a measure of transmural dispersion of repolarization which can be used as a tool for detection of life threatening arrhythmias [5].

Only a few studies have demonstrated these repolarization changes after the acute phase of illness. The long term impact on myocardial electrical stability after KD is not well understood especially in children with no cardiac involvement during the acute phase. So a prospective cohort study was conducted on 20 children with history of KD for the analysis of repolarization changes on ECG.

## 2. Methods

A hospital-based prospective study was conducted at a tertiary care centre between October 2014 and September 2016. Children with history of KD diagnosed at least 3 months prior were included in the study. Patients who fulfilled the inclusion criteria were recruited into the study after getting clearance from the institutional ethics committee. Informed consent was obtained from either of the parents of the child and a semistructured proforma was prepared to record all data.

A 12-lead ECG was obtained from each subject after a period of 5 minutes of rest using CARDIART 6108T ECG system at 25 mm/sec and 50 mm/sec paper speed. Measurements were made manually using STANDARD DIGITAL CALIPERS (AEROSPACE, China). The following variables were measured using the calipers

1. Qt dispersion (QTd): defined as the difference between maximum and minimum QT interval of a 12-lead ECG.
2. T peak to T end (Tpe) interval: the peak of the T-wave as defined as a point of highest amplitude of the T-wave deflection and the end as a point where the tangent on descending limb of T-wave intersects the isoelectric line.
3. T peak to T end/QT(Tpe/QT) ratio.

ECGs with low amplitude or unreliable T-waves and U waves were excluded from analysis. The mean QTd were calculated from all the 12 leads in the study group and compared with the control group. Tpe and Tpe/QT ratio were measured in one of the limb leads (II) and chest leads (V5) and the mean calculated values were compared between the groups.

Blood pressure was recorded for all the children included in the study using a standard mercury sphygmomanometer with appropriate cuff size. A repeat ECG was obtained from the study group after a period of 9 ± 2 months and the same variables were measured.

ECG was interpreted with the guidance of a cardiologist with fellowship in cardiac electrophysiology and a 2D ECHO was performed on all children with history of KD by the same cardiologist to look for coronary artery abnormalities. Out of the 696 leads obtained, 71 leads had to be excluded from analysis because of the poor T-wave formation and presence of U waves.

Collected data were analyzed using statistical package for social sciences (SPSS) version 17.0. The data was presented as mean and standard deviations. Statistical analysis for difference in mean value was performed using student *t*-test. Correlations were analyzed by analysis of variance (ANOVA). *P* value of less than 0.05 was considered significant.

## 3. Results

In children with history of Kawasaki disease, QT dispersion is significantly increased compared to the control group. The mean value of QTd in the first ECG in children with KD was 43.89 ± 14.529 msec when compared to 29.47 ± 8.637 msec in the control group.

**Figure 1:**
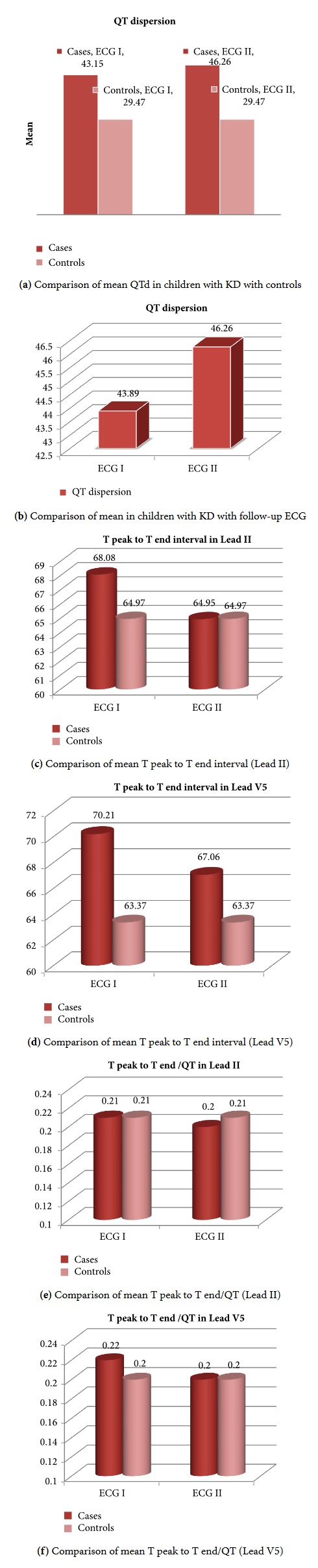


The follow-up ECG also showed similar result with a mean value of 46.26 ± 16.254 msec in the study group when compared to the control group (Figure 1(a)). QT dispersions were increased in the follow-up ECG when compared to the first ECG in the control group but are not statistically significant (Figure 1(b)).

There was no statistical significance observed in the Tp-Te interval in cases when compared to controls, Lead II (68.08 ± 17.11 versus 64.97 ± 14.185; *p* = 0.099), and Lead V5 (70.21 ± 15.293 versus 63.37 ± 10.877; *p* = 0.509) (Figures 1(c) and 1(d)). The same was observed with Tp-Te/QT ratio in cases compared to controls (Lead II- 0.21 ± 0.05 versus 0.21 ± 0.034; *p* = 0.138; Lead V5- 0.22 ± 0.05 versus 0.20 ± 0.031; *p* = 0.734 (Figures 1(e) and 1(f)). The follow-up ECG in cases also showed no statistical difference in the Tp-Te interval and Te/QT ratio.

## 4. Discussion

Kawasaki disease (KD) is an acute, self-limiting, febrile illness that occurs predominantly in children with pathology demonstrating vasculitis of small and medium size blood vessels with a predilection for coronary arteries. It is complicated by either clinical or subclinical myocarditis in the acute stage and can lead to histological changes of the myocardium such as interstitial fibrosis.

We attempted to study the hypothesis that, in children with KD without overt coronary artery involvement, myocardial abnormalities may persist even after acute phase which is reflected on surface ECG as repolarization changes. The study was conducted on 20 children with history of typical KD without coronary artery involvement in the acute phase and treated with 2 g/kg of IVIG within 10 days of onset of illness (diagnosed based on American Heart Association (AHA) criteria) and an equal number of age and sex-matched controls.

QT interval dispersion (QTd) represents a general abnormality in repolarization. Increased QT dispersion has been demonstrated in various cardiac diseases. Data obtained from almost 7000 patients with cardiac disorders like myocardial infarction, cardiomyopathies, and long QT syndrome have shown that there is definite evidence of increased QT dispersion in these cardiac disorders [6].

Our study showed significant difference in the QT dispersion in children with KD when compared to the control group. The follow-up ECG which was done 9 ± 2 months showed increased QT interval dispersion compared to the initial ECG, but the increase was not statistically significant.

In the year 1999, a study was conducted by Osada et al. [7] wherein they have studied the QTd. Children with KD were grouped based on the degree of involvement of coronaries during the acute phase. It was found that QT interval dispersions were proportionately increased with the degree of involvement of coronaries. The main drawback of the study was lack of control group to compare the results.

Three years later Dahdah et al. [3] conducted a similar study. Children with history of KD were categorized into three different groups not just based on the degree of involvement of coronaries but depending on the degree of resolution of coronary aneurysms. The study concluded that QTd and QTcd (corrected Qt interval dispersion) were increased in patients who had persistent coronary artery aneurysm.

In 2008, Ghelani et al. [8] performed a study on Indian population which included children with history of KD with no overt coronary artery abnormalities. QTd was measured and compared with the control group. The study showed significant increase in QTd when compared to the control group thereby concluding that there is evidence of heterogeneous ventricular repolarization in children with KD who had no apparent coronary involvement during the acute phase.

Similarly, in 2014, Gupta et al. conducted a study on a cohort including 30 children with history of KD and measured QTd. Though it was not the main objective of the study they have concluded that study population showed increased QTd when compared to the control group.

Recently a study conducted by Parihar et al. [9] on a cohort of 20 children diagnosed to have KD with transient coronary artery abnormalities during the acute phase concluded that there is no significant increase in QTd which is contradictory to the above-mentioned studies.

### 4.1. T Peak to T End and T Peak to T End/QT

Apart from QT dispersion, we have also studied the ventricular repolarization changes, namely, T peak to T end interval and T peak to T end/QT ratio. These variables have been used extensively in predicting the arrhythmic risk in various cardiac disorders like myocardial infarction, hypertrophic cardiomyopathy, and pulmonary embolism (PE) [10]. It has been proposed that Tpe and Tpe/QT variables represent a more precise tool for measuring the ventricular repolarization as these variables are less dependent on heart rate, autonomic changes, and QRS duration [11].

Evidence supporting these parameters as a prognostic tool in forecasting the risk of arrhythmias have been provided under congenital and acquired long Qt, hypertrophic cardiomyopathy, and Brugada syndrome [12]. A systematic review and meta-analysis also concluded that T peak to T end interval is a useful risk stratification tool in various cardiac illnesses [13].

In a study done by Abdullah Icli et al. [14] it was proposed that Tpe interval can be used as a prognostic tool as risk stratification in patients with acute PE. A study conducted by Hamid Amoozar et al. [15] on 30 children with KD, concluded that Qt interval and T peak to T end interval were significantly increased during the acute phase of illness. In another study conducted by Masayuki Fujino, it was concluded that Tpe/QT ratio is useful for evaluating severity of vasculitis and myocarditis associated with KD [16].

The ratio between the Tpe interval and QT interval (Tpe/QT) has been proposed as a noninvasive marker of arrhythmic risk along with Tpe interval, while QT interval and Tpe interval vary with various body sizes Tpe/QT remains relatively constant. It was also reported to be stable with varied heart rates [17]. Our study showed no significant changes in these variables in the study group when compared to the control group.

## 5. Conclusion

There is evidence of repolarization changes in the myocardium in children with history of KD without cardiac involvement in acute stage even after treatment with IVIG/Aspirin in recommended doses. This cohort may be at risk of developing long term complications like arrhythmias. This cohort requires long term assessment and follow-up to identify the risk of myocardial dysfunction. Further studies are warranted on a larger sample size to identify the significance of these repolarization parameters in forecasting the arrhythmic risk.

## Data Availability

The data used to support the findings of this study are available from the corresponding author upon request.
